# Impact of COVID-19 on search in an organisation

**DOI:** 10.1177/0165551521989531

**Published:** 2023-02

**Authors:** Paul H Cleverley, Fionnuala Cousins, Simon Burnett

**Affiliations:** Robert Gordon University, UK

**Keywords:** Coronavirus, enterprise search, search behaviour, uncertainty

## Abstract

COVID-19 has created unprecedented organisational challenges, yet no study has examined the impact on information search. A case study in a knowledge-intensive organisation was undertaken on 2.5 million search queries during the pandemic. A surge of unique users and COVID-19 search queries in March 2020 may equate to ‘peak uncertainty and activity’, demonstrating the importance of corporate search engines in times of crisis. Search volumes dropped 24% after lockdowns; an ‘L-shaped’ recovery may be a surrogate for business activity. COVID-19 search queries transitioned from awareness, to impact, strategy, response and ways of working that may influence future search design. Low click through rates imply some information needs were not met and searches on mental health increased. In extreme situations (i.e. a pandemic), companies may need to move faster, monitoring and exploiting their enterprise search logs in real time as these reflect uncertainty and anxiety that may exist in the enterprise.

## 1. Introduction

In order to find information more efficiently, many organisations such as the NHS, UNICEF, HP, AMN Healthcare, NASA, The World Bank, AstraZeneca, BP, Boeing, US Library of Congress and NATO [1] have invested in Enterprise Search technologies. The corporate ‘Google’ enables staff to search and exploit their organisation’s distributed information repositories (such as their Intranet and documents). Search engines have become so prevalent in everyday use they have become an epistemology in places – how we come to know things, so are of significant research importance [2].

Due to difficulties accessing primary data, there are few context bound sociotechnical studies on search in the enterprise [3,4]. Where searching for information in the workplace is situated in an ecosystem not a vacuum [5–7].

Using search log data as ‘digital body language’ [8], there is emerging evidence [9,10] showing the COVID-19 pandemic [11] has significantly impacted Internet consumer and scholarly information search behaviour. Governments have even used Google searches as part of ‘syndromic surveillance’ to assess societal impact [12,13].

It has also been suggested that health crises are also information crises, with a call for more research into information behaviours and environments during global health crises [14]. While there are studies investigating the role of information technology (IT) in general to COVID-19 organisational impact [15], there are no known studies that have examined how a pandemic, disaster, security lockdown or public health restrictions have specifically impacted search behaviour in the organisation. This provides the rationale for this study. The following section reviews the academic and practitioner literature and introduces the research questions.

## 2. Literature review

The literature review will cover information searching, followed by enterprise search and then discuss the literature on analysing Internet search behaviour during pandemics.

### 2.1. Information searching

People often seek information to resolve some level of uncertainty. The information search process has been described as thoughts, feeling and actions as people seek meaning as they seek information; uncertain thoughts, doubts and anxiety, typically (but not always) becoming clearer, more specific, and more confident as the search process evolves [16]. These models can help interpret behaviour associated with certain patterns observed in the search transaction logs of search engines.

Six intents for consumer Internet search have also been proposed, ‘surprise me’, ‘thrill me’, ‘impress me’, ‘educate me’, ‘reassure me’ and ‘help me’ [17]. It is probable that some of these such as ‘reassure me’, ‘educate me’ and ‘help me’ are more relevant in the enterprise than others, such as ‘thrill me’.

Two main search goals have been identified, lookup/known item where there is a right answer and exploratory search where the goal is more focused on learning and there is no single right answer to be returned by the search engine [18]. The transition from exploratory browsing to lookup focused searching may involve a ‘eureka’ moment [19].

For tactics when searching information, numerous strategies have been documented [20] such as broadening and narrowing [21], where narrowing is typically achieved by adding more search query terms. A space between search query terms is almost universally accepted as some type of Boolean AND logic. Some users often make numerous queries to cater for the fact that the same concept can be described using different terminology, what is termed the vocabulary problem [22].

Moving away from a ‘user’ focused position, Human Information Interaction [6] has been described as the area of study that describes how humans interact with information, with actors rather than users in a system constrained by social and environmental contextual factors that influence search behaviour. This recognises that people help construct (agency) their knowledge, culture and institutions and are changed by them (structure) at the same time akin to recursive, self-referential feedback/mutual causality co-evolutionary processes [23–25]. Social phenomena such as changes in search behaviour in the organisation are therefore not the product of agency or structure, but both.

### 2.2. Business continuity and enterprise search

The 9/11 terrorist attack forced many businesses to evaluate their business continuity plans to remain commercially operational in exceptional circumstances [26]. Where businesses must consider disruption on three resource types: people, information and technology. Morgan Stanley reduced the anxiety levels of staff during the 2003 SARS pandemic, including daily webcasts and dedicated pages of information on the Company Intranet [27]. During the H1N1 (swine flu) outbreak in Singapore [28], mass media (television, newspapers and radio) were the most heavily used sources of information along with networks of relatives and friends for prevention, treatment and to reduce fear. It has been reported that online information sources were relatively minimal with school, news websites (e.g. BBC) and the Company Intranet in the top three. This contrasts from other studies [29] that show the Internet was the overwhelming source of information for swine flu, which may reflect different community sampling or information obtainability [30].

A series of events were taken by a Brussels bank during November 2015 [31], where lockdowns were initiated while terrorists were at large. The situation became chaotic, the mobile phone network collapsed and ‘every staff member went online on the Internet. Official information and guidance was quite blurred, while hastily published press releases contradicted each other’ [31]. On the Sunday before lockdowns, when many staff worked remotely from home, an increase in users was reported in the search logs looking for more information on the Company Intranet.

However, it has been reported that most organisations do not have the resources to look at their search logs [32]. Enterprise search queries are short [32,33], and some studies reporting 79% of all queries are two terms or fewer [34]. It has been found that 80% of users tended to use the search system and immediately stop, termed ‘casual unsophisticated users’ which may be related to lookup/known item ‘fact seekers’ and people using search for ‘bookmarking’ [35]. Similar patterns are found in libraries [36] and in other organisations, termed ‘hit and run’ [37].

In analysis of corporate search log data, ‘long and varied’ user groups (between 13% and 24%) have been identified that include infrequent searchers [37]. This may represent exploratory search or struggling lookup search sessions [38]. This is supported by other studies that identify 20% of users who had much longer sessions, made more queries and spent more time examining documents [35]. These were termed ‘knowledgeable’ and ‘intensive’ users who may prefer recall over precision. Combining and synthesising the literature [2,7,18,32,33,35,37,38,39], Table 1 shows the typical work tasks and related information tasks serviced by enterprise search as a ‘one size fits all’ information system.

**Table 1.** Typical work activities supported by enterprise search.

[35,37,38]	80% ‘Fact Seekers’, ‘Hit and Run’ users				20% ‘Knowledgeable’ and ‘Intensive’ users
[7]	Structured (instructional)				Unstructured (constrained)
[18]	Lookup/known item search				Exploratory search
[39]	Transactional	Navigational	Informational		
	Locate and launch application	Visit website	Open document	Provide answer	Browse, summarise information
ADMINISTRATIVE (e.g. time-writing, expenses, travel, training)	HIGH VOLUME/RELEVANCE				LOW VOLUME/RELEVANCE
KNOWLEDGE INTENSIVE (e.g. ideation, analysis, decision-making)	LOW VOLUME/RELEVANCE				HIGH VOLUME/RELEVANCE

While enterprise search logs have been studied to infer information behaviour, there are gaps in the literature relating to the impact that an extended crisis has, such as a pandemic, on information behaviour.

### 2.3. Internet search patterns and pandemics

Internet search engine data have been used for surveillance for previous pandemics, such as H1N1 [40] and Ebola [41]. They note, however, that for well-publicised diseases, many spikes were driven by publicity rather than the disease itself. The conclusion was that for diseases with considerable media exposure, using Internet search patterns for prediction can be misleading. The Google Flu Trends algorithm [42] was designed to predict outbreaks of influenza (flu) from Google web searches 2 weeks before traditional methods, termed ‘infodemiology’. However, it was cancelled in 2015 because it did not predict the non-seasonal 2009 H1N1 pandemic with opaque computational methods [43].

Many researchers, however, are revisiting the predictive use of search query data when used in combination with other methods [44]. It has been reported that while some search trends for COVID-19 were due to media coverage, other clinical manifestations including shortness of breath, headache, chest pain and sneezing showed strong correlations with real-world cases and deaths [45]. Google Trends has been used to show a statistically significant correlation between ‘loss of smell’ related search query volumes in numerous countries with daily confirmed cases of COVID-19 [46]. They postulate that it may refer to a previously unrecognised symptom. It has been suggested that monitoring web queries for risk management strategies such as ‘hand washing’ and ‘face masks’ could help make appropriate communication interventions [47].

Analysis of worldwide COVID-19 web queries using Google Trends [48], found 12 March was the peak in query volumes, the day after the World Health Organisation declared COVID-19 as a pandemic [11]. However, Google Trends data indicate the (search frequency) peak was the 16 March 2020. Search queries can potentially represent what is ‘on people’s mind’s’. Google Trends has been used to analyse web searches in Europe and America during the COVID-19 lockdown with indications that people’s mental health may have been severely impacted [49]. While insights are being made between search behaviour and the COVID-19 pandemic from Google, there are no studies that examine the impact on searching in the enterprise.

**Figure 1.**
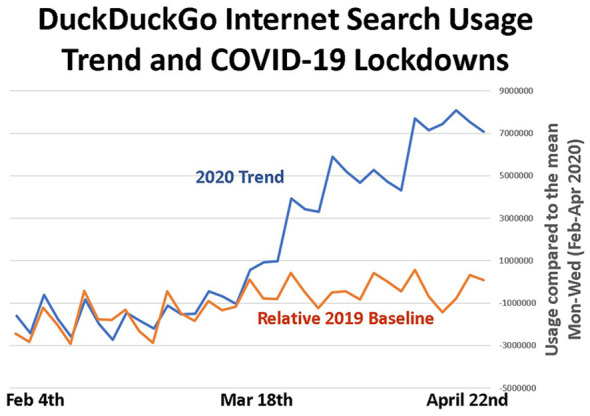
Growth in Internet search usage [9]. Copyright, reprinted by permission www.paulhcleverley.com.

There is evidence that usage of Internet search has grown exponentially during lockdowns [9]. As shown in Figure 1 between February and April 2020, usage volumes compared with the mean values for that period show a steep increase in search usage from around 18 March onwards.

**Figure 2.**
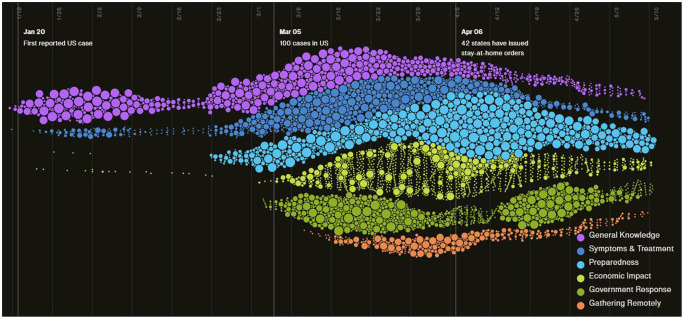
COVID-19 Google Trends Beeswarm chart: January to April 2020 [52]. Copyright, reprinted with permission https://searchingcovid19.com/.

This roughly coincides with numerous governmental restrictions and lockdowns caused by COVID-19 and are not present on the 2019 seasonal baseline. These data appear to provide support for an ‘exponential growth’ pattern of Internet search engine use while more people are at home during lockdowns, a trend reported by others [50]. The average Click Through Rate (CTR) for Google has been reported at 66% [51], representing the proportion of search queries that lead to a user clicking on a search result. The Google Trends application programming interface (API) has been used [52] to analyse thousands of Google COVID-19-related queries made in the United States, restricting queries to ‘How to …’ and ‘What is/are …’ (Figure 2).

In Figure 2, each bubble represents a top 10 trending search query in one or more US states, the size of bubble is prevalence across US states. Initial intents started with ‘What is/are …’, such as ‘What are the symptoms of coronavirus?’ transitioned to include ‘How to …’ such as ‘How to make a face mask with fabric?’, which may represent a tendency to move from exploratory to lookup search tasks. Asking questions of search and conversational assistants has grown in popularity, representing 8% of all Google queries [51].

**Figure 3.**
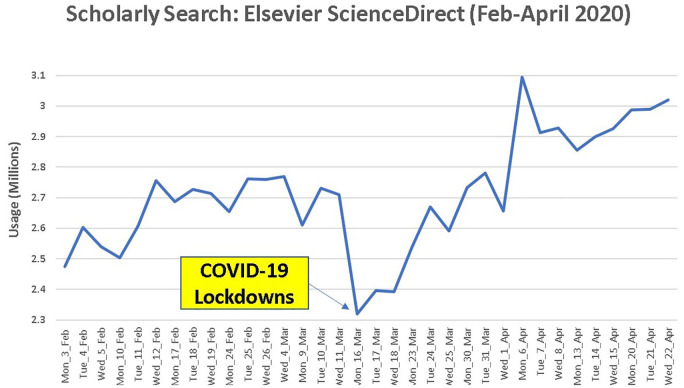
‘V’ shaped recovery in Elsevier Scholarly Search [9]. Copyright, reprinted with permission from www.paulhcleverley.com.

Search volumes for scholarly search have been examined during the COVID-19 pandemic [9]. Figure 3 shows the 16 March coincides with Government announcements on restrictions, representing a major fall in usage and ‘V’ shaped rebound.

While it is clear there are differences in search behaviour during the pandemic between Internet and Scholarly search environments, there have been no comparisons with the enterprise environment. There are no known studies showing how enterprise search inside an organisation has been affected by COVID-19. The literature review has identified a gap on how usage patterns in enterprise search may have been affected by COVID-19 and what may be on people’s minds in an organisational context. These lead to the following research questions:

*RQ1.* To what extent has COVID-19 affected enterprise search usage?
*RQ2*. What insights can be inferred from COVID-19-related search queries in an enterprise?

## 3. Methodology

### 3.1. Philosophy

It has been suggested that a gap needs to be bridged in information science between professional business practice and research [53]. Some scholars have already taken a position that much information science research needs to expand and become more relevant to practice [54,55]. A constructivist philosophy, pure description of search behaviour, is unlikely to answer the research questions and be of little practical use to organisations. Conversely a purely positivist approach, just testing one variable against another does not offer any causal explanation; it does not answer the question why or how something occurs [56].

Critical realism recognises the complexity of social phenomena, combining methods where plausible explanations are the goal, using judgmental rationality to compare and assess competing theories based on their explanatory adequacy or power [57]. Critical realism is seen as a compelling ‘third way’ [58], and for these reasons, it is adopted as the philosophy for this study.

### 3.2. Research strategy

A case study [59] was chosen as an appropriate research strategy to gain deep insights through time (longitudinal) for enterprise search user engagement and search queries. A large knowledge-intensive multinational corporation with over 100,000 staff (majority office based) was chosen as it gained exposure to multiple countries affected by the pandemic and provided enough data for meaningful analysis over the study period, with little/no staff furloughed. The search index contained several hundred million items including Intranet web pages, office documents and discussion threads. The organisation is to remain anonymous to avoid recognition by peers, competitors and stakeholders.

### 3.3. Data collection

Over 2.5 million queries from search transaction log data were collected from the enterprise search engine as a way of sampling the entire organisation to assess search behaviour changes through time. With COVID-19 lockdowns in many countries starting in early/mid-March, a study time period of the beginning of January to the end of May 2020 was chosen. This would most probably supply two balanced time periods before and after major country lockdowns while also encompassing the Wuhan lockdown in China that took place in January 2020 [11]. For a baseline, 2019 data were also collated to rule out changes that may be seasonal.

The data collected consisted of the actual search queries made, their frequency, date and CTR, representing the percentage of those queries made where a user clicked on a result. The rationale being the lower the CTR, the more probable that users making that query did not see any results that they deemed useful to click on. Data on the total unique users per day and total search queries made per day (including how many were pagination/scrolling queries) were also collected.

There were certain limitations on the search log data collated by the organisation. All usage data were anonymous to the individual, but no unique identifier was used, making it impossible to link sessions. It was therefore not possible to track individual user behaviour changes.

External news reports and internal company communications were also part of the data collation, creating a timeline of events for COVID-19 that may explicate any patterns observed in the search log data. Where appropriate, questions were asked to the enterprise search service team when clarifications were needed.

*RQ1*. To what extent has COVID-19 affected enterprise search usage?

Three parameters –*search query volumes per day, number of unique users per day* and *average number of queries per user per day*– were selected to investigate how COVID-19 may have affected enterprise search user engagement and search behaviour. The literature has previously described how Internet and scholarly search have been affected with similar parameters [9]. The classic search log graphic of usage over time is a ‘saw tooth’ with peaks of usage corresponding to workdays and much lower usage at weekends and holidays. To ensure visual patterns were not impaired, Friday, Saturday and Sunday along with major holidays were removed in 2020 and 2019 data for aesthetics where appropriate. The 2019 data were also shifted by a day where appropriate to ensure weekdays ‘lined up’ allowing like for like comparison.

*RQ2*. What insights can be inferred from COVID-19-related search queries in an enterprise?

Ensuring the search queries used were related to COVID-19 is challenging, to avoid issues of using search query terms not uniquely related to the pandemic that may lead to erroneous conclusions [43]. This is particularly challenging in enterprise search when the average number of words used in a search query is lower than that used in Internet search engines [34] so judging user intent may be prone to error. For these reasons, only search queries containing an obvious synonym for COVID-19 were counted per day. The parameter was termed *explicit COVID-19 search query volumes per day*. Over 2500 explicit COVID-19 search queries were identified. This would most probably underestimate the total volume of queries made relating to COVID-19, but would ensure precision of data collection. In addition, the more obvious implicit search queries related to areas such as symptoms, health and new ways of working were analysed in the search log data, but not included in the frequency counts.

Names used for COVID-19 were inductively identified from the data (rather than having a pre-defined list) to minimise missing information. Only queries made eight or more times were counted to limit the volume of data analysed. These queries were further split based on the number of words used in the search query. Where the terms ‘covid 19’ or ‘corona virus’ were used in the search query, these were treated as single-word (concept) queries. Search queries such as ‘cognitive behavioural therapy covid19’ were treated as a four-word query, for example. Many of the search queries are sensitive and could lead to the identification of the organisation, so cannot be reported or were redacted in part.

### 3.4. Analysis

Thematic mapping was undertaken [59] to group explicit COVID-19 search queries into categories guided by the categories used in previous COVID-19 search analysis [52]. To test statistical significance, an independent (unpaired) two-tailed *t*-test was used to compare the mean values of given parameters in the search log populations ‘before’ (population #1) and ‘after’ (population #2) major gradient changes in search log usage patterns. The parameters *search query volumes per day, number of unique users per day, average number of queries per user per day* and *number of search terms in explicit COVID-19 search query volumes per day* were analysed. The *t*-test measures the differences in the mean values within both groups, to ascertain if the differences are statistically significant; in other words, whether they could have occurred by chance [60]. A *p*-value of <0.05 is deemed statistically significant for this study. These were also conducted on 2019 data over the same time period to test if any differences could be due to seasonal effects.

## 4. Results and discussion

The results are presented in this section immediately followed in-turn by a discussion to aid understanding and flow for the reader.

### 4.1. Effect of COVID-19 on enterprise search usage

**Figure 4.**
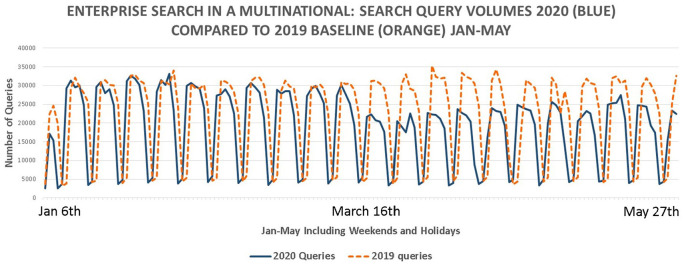
Enterprise search query volumes per day (January to May). Italy announced lockdowns on 9 March, France on 17 March, the United Kingdom advised working from home and non-essential contact on 16 March and lockdowns on 23 March. The United States announced travel bans from Europe on 15 March. By 27 May, most lockdowns were still in place with few changes.

Search query volumes per day are shown in Figure 4 for 2020 (solid lines) with a 2019 baseline (dotted) including weekends and holidays. The classic ‘saw tooth’ shape is visible (Figure 4).

The initial 24% decrease in search query volumes from 16 March onwards in 2020 can be seen in Figure 4. By the end of May, search query volumes are still to reach pre-16 March levels at 13% lower. Search volumes post-16 March are lower than those prior to 16 March *t*(71) = 2.25, *p* < 0.05, which is statistically significant. No such difference exists in the 2019 baseline, *t*(71) = −0.096, *p* > 0.05. It is therefore unlikely that seasonal changes (such as Easter) can explain the significant drop in search volumes. Visiting the literature and news [11], the timing suggests COVID-19 lockdowns may be a causal factor.

The usage volumes from Figure 4 show a significantly different pattern to that of the ‘exponentially growing’ Internet consumer search usage volumes (Figure 1) and the ‘V-shaped recovery’ of scholarly search (Figure 3). Enterprise search data in this case study point to a large drop in usage and hint of a possible ‘L-shaped recovery’ as the initial drop in search volumes from 16 March (−24%) has gradually increased, although still short (−13%) of pre-lockdown levels by 27 May.

Approximately, 20% of all queries were related to pagination/scrolling, which remains the same as a percentage both before and after 16 March. This may support existing studies [35] indicating 80% of enterprise search users are probable to be casual unsophisticated users (do not click past page 1), termed ‘hit and run’ [37] and may be independent of environmental changes.

**Figure 5.**
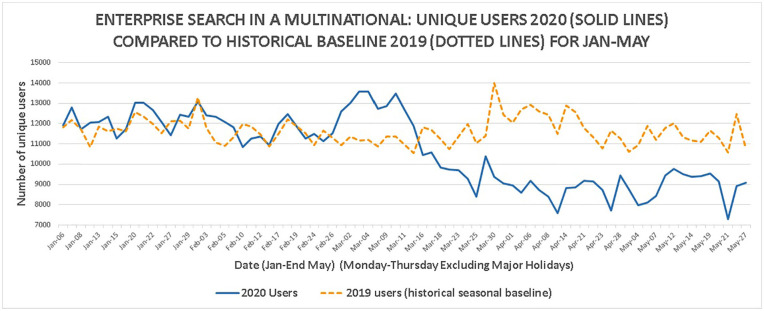
Enterprise search unique users per day (January to May).

The number of unique users per day is shown in Figure 5 with weekends, Fridays and holidays removed.

Between 2 March and 12 March 2020, the ‘twin peaks’ (Figure 5) show a surge in unique users before dropping off during the week of 16 March and not recovering to prior levels, remaining around 25% lower. The timing broadly matches (but slightly earlier) that the web search trends analysis [48] who reported that 12 March was the ‘peak’ for search query volumes on COVID-19.

For 2020, the number of unique users after 16 March is lower than the number of unique users before 16 March *t*(71) = 2.76, *p* < 0.05 which is statistically significant. There was no statistical difference for the 2019 data *t*(71) = 0.00739, *p* > 0.05. It is therefore unlikely that seasonal effects are responsible for the decrease in unique users. Triangulating with the data from Figure 4 and external news accounts [11], the most plausible explanation is the COVID-19 pandemic being a causal factor.

The enterprise search unique users per day ‘twin peaks’ (2 March to 12 March, Figure 5) are an interesting pattern that warrants more discussion. Unique users peaked at 13,564 in the period. The unique number of users in the 2020 peak exceeded 2019 levels (by over 13%). This could be related to people searching to meet a range of tasks (including administrative in Table 1) before anticipated remote working or lockdowns occur – a surge in business activity.

Another explanation may be related to ‘peak uncertainty’ [16,30] where staff attempted to allay their thoughts of uncertainty regarding the pandemic before the organisation made announcements about working from home on 13 March and before governments made COVID-19 announcements during the week beginning 16 March [12]. This information behaviour may be similar to the increase in Intranet search users the day before the lockdowns during the Brussels terrorist incidents [31]. The sharp decrease in unique users may represent in some part a reduction in uncertain thoughts as the enterprise communicated its approach to staff regarding lockdowns.

Investigating the queries made in the search log during this time, on 2 March and 10 March, there are spikes in queries for administrative tasks (Table 1). This period also sees spikes in explicit COVID-19 queries (see later, Figure 7). Given this, the most plausible explanation is probable to be a combination of these two factors, which shows the importance of an effective working search engine during a crisis to support increased business activity and help mitigate uncertainty and concerns among staff.

The search volumes and unique user counts have not bounced back after the initial drop in lockdowns, unlike Internet search and scholarly search [9]. This could be related to cuts the organisation has made to projects and its workforce (like many organisations); these may have led to a reduction in work activities and therefore usage of enterprise search, reflecting a holding pattern between basic business continuity and actual business as usual in a ‘new normal’. In this explanation, search volumes and unique users could be a surrogate for levels of business activity. Another explanation is that technology and virtual private network (VPN) network connection problems from home may have caused problems for some (a potential barrier) using the search engine outside the physical workplace.

**Figure 6.**
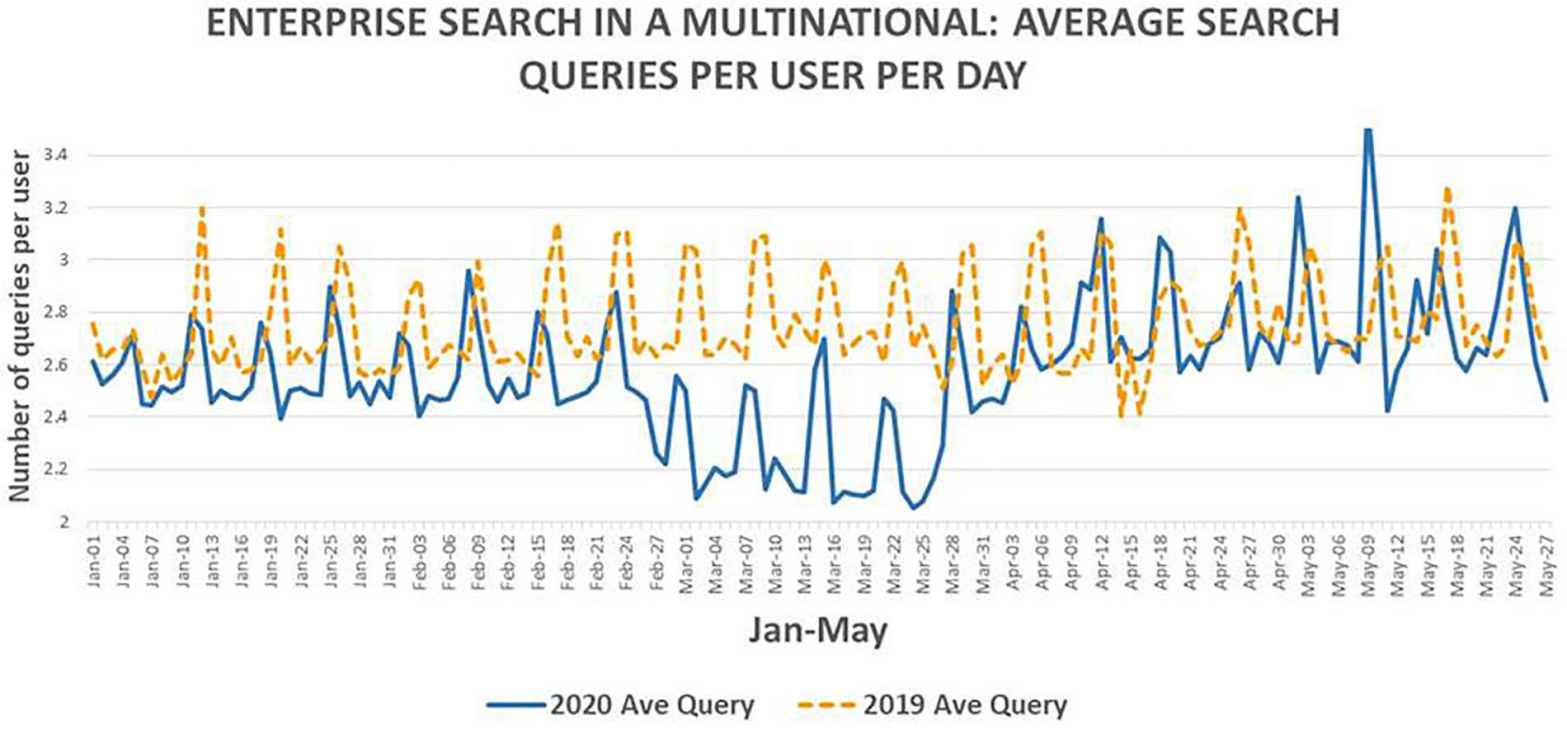
Average number of search queries per user per day (January to May).

The former explanation seems more plausible being so consistent over an extended period while still supporting thousands of users, although there may be effects of poor IT connectivity at home at times. Dividing the number of non-paginated search queries per day by the number of unique users allows the computation of average number of search queries per user per day. This is shown in Figure 6.

The average number of queries per user per day shows an interesting ‘U’ shaped drop in 2020 between 24 February and 28 March (Figure 6). It is possible that this ‘U shape’ is related to two causal factors: first, an influx of casual unsophisticated users [35] during the first part of the period coinciding with the surge of unique users shown in Figure 5, which has the effect of reducing average search queries per user per day; second, an overall drop in knowledgeable intensive users [35] in the second part of this period caused by disruption as staff started working remotely from home in earnest and reduced project work and business activity. This would also have had the effect of reducing average search queries per user per day. This seems the most plausible explanation for this month long ‘U’ shape pattern for average search queries per user per day.

The average search queries per user after 28 March are higher than those before 28 February, *t*(60) = −5.323, *p* < 0.05 which is statistically significant. However, this trend as also seen in the 2019 data *t*(60) = −2.049, *p* < 0.05. It appears that in April/May, users conduct more individual queries than in January/February as a potential seasonal effect. The COVID-19 pandemic may therefore not be a major causal factor for this difference.

In summary, the COVID-19 pandemic has had a significant impact on enterprise search usage that appears unprecedented in recent times. Statistically significant decreases in search volumes and unique users are seen after 16 March that are not seasonal, which appear to be following an ‘L-shaped’ recovery. These differ from Internet and scholarly search patterns during the pandemic. A surge in unique users just before 16 March appears related to completing work tasks before lockdowns and allaying concerns and uncertainty about COVID-19.

### 4.2. Inferences from COVID-19-related queries in enterprise search logs

**Figure 7.**
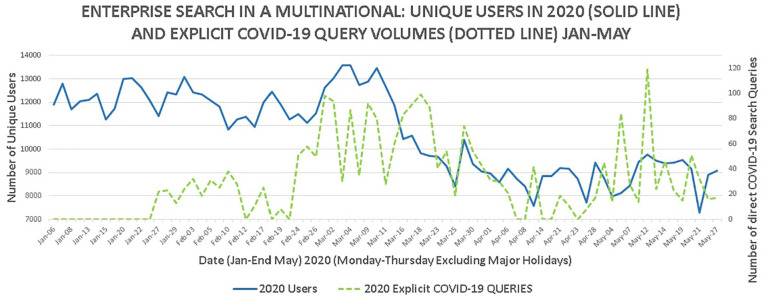
Explicit COVID-19 search queries and enterprise search unique users.

It was confirmed in June 2020 by the IT group managing the enterprise search engine that the search log data have not been used by the organisation during the COVID-19 pandemic. The number of enterprise search unique users in 2020 (from Figure 5) is shown in Figure 7 (solid line) along with the volume of explicit COVID-19 search queries (dotted line).

Nine variants of single-word/concept COVID-19 search queries were identified: ‘Coronovirus’, ‘corona virus’, ‘covid’, ‘coronavirus’, ‘corona’, ‘covid-19’, ‘covid19’, ‘covix-19’ and ‘covid 19’. The terms ‘SARS CoV2’ or ‘2019-nCov’ were not seen in the search logs. A further 45 unique multi-word queries were identified part containing these terms (such as ‘fatigue covid’ and ‘cognitive behavioural therapy covid19’).

The first major occurrences of explicit COVID-19 queries occurred around 27 January 2020, probably related to the Wuhan (China) lockdown [11]. The frequency of the queries waned until 27 February to 2 March which saw a spike in search queries related to COVID-19. This appeared to coincide with the surge of unique users (see Figure 7). Subsequently, the number of queries reduced, spiking again in mid-May.

**Figure 8.**
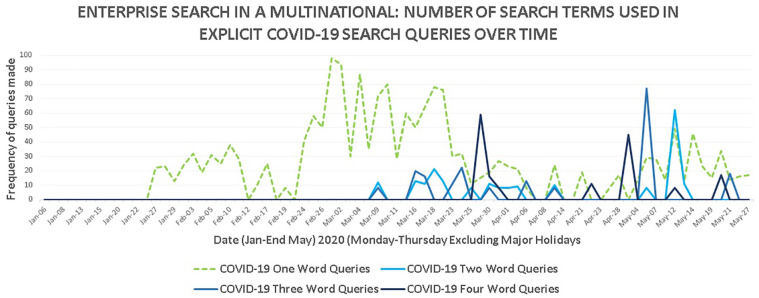
Number of search terms used in explicit COVID-19 search queries.

The explicit COVID-19 search queries are direct indicators of information intent from staff to seek COVID-19 information. This shows the importance of the enterprise search engine to meet information needs in a crisis. The explicit COVID-19 queries were further subdivided by the number of words in search query terms, shown in Figure 8.

A striking pattern is observed (Figure 8) showing that explicit COVID-19 search queries prior to 16 March were almost exclusively single word (dotted lines). Subsequently, multi-word queries (up to seven-word queries) became equally as important by volume as intents narrowed. The number of words used in an explicit COVID-19 search query after 16 March increased compared with before 16 March *t*(36) = 0.0091, *p* < 0.05 which is statistically significant.

We therefore infer there is a difference between how people searched explicitly for COVID-19 information running up to lockdown versus post-lockdown. This shows a transition from broad, single-word exploratory like explicit COVID-19 queries which may be driven by intents such as ‘reassure me’ and ‘educate me’ [17] to narrower task-driven queries related to safety, business impact (such as ‘covid impact to supply chain’), strategy, policy and response. This follows the information search process [16], uncertain thoughts becoming more specific; however, this relates to the staff community ‘as a whole’ not just individuals.

No explicit COVID-19 queries that included questions – such as ‘What is …’‘How to …’– were found in enterprise search logs. One explanation is adaptation, where users in the enterprise have learnt how their classic keyword search engine behaves compared with sophisticated Question and Answer systems such as Google [51]. Users predominantly use nouns in the enterprise search, while search queries framed as a question, make up <0.5% of all queries made in the enterprise search in the case study organisation. This is substantially less than Google where 8% of all search queries are framed as questions [51]. Information search behaviour in the case study organisation has therefore probably been shaped somewhat by culture (the technology artefact).

People were also most probably looking for specific documents to support work tasks related to COVID-19 such as ‘covid 19 aviation strategy’ with information on strategy probable to be represented in some form of document, rather than an answer/fact or text on a web page.

**Figure 9.**
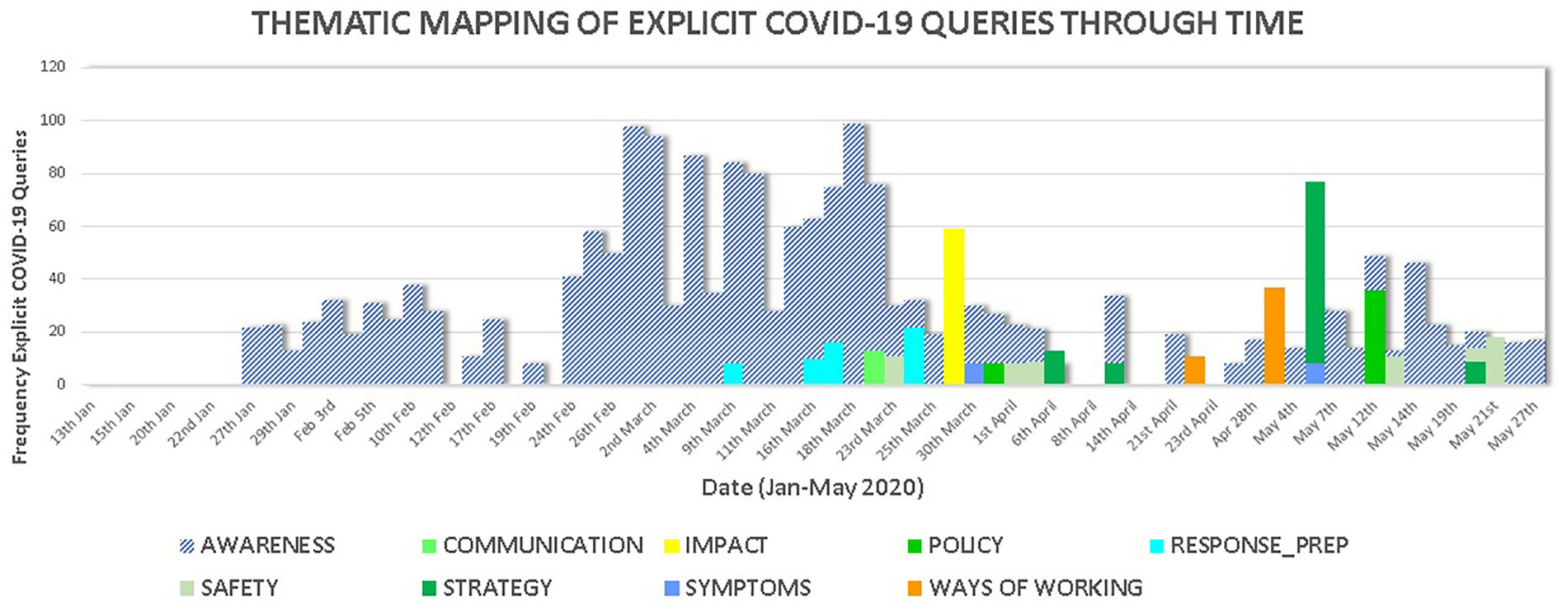
Thematic mapping of COVID-19 enterprise search queries through time.

The explicit COVID-19 search queries (equalling or subsuming one of the nine explicit COVID-19 terms described earlier in this section) broken down in Figure 8 by number of words in the search query were grouped into thematic categories (Figure 9). The purpose was to compare the patterns with the sequence shown in Figure 2 from the literature review, to elicit any insights.

The same coding was used where possible, to that used by [52] in Figure 2. Similar sequencing and overlapping patterns exist, with early single-word search queries probably having a broad awareness intent. There are three groupings seen from January to May 2020.

First (left-hand side of Figure 9), late January to middle February search queries were low in frequency and awareness based. Second (middle of Figure 9), late February to middle April shows an initial peak in frequency of awareness-based search queries followed by search queries related to communications, response and impact assessments. Third (right-hand side of Figure 9), late April to end May shows search queries focused on ways of working, strategy and policy.

**Figure 10.**
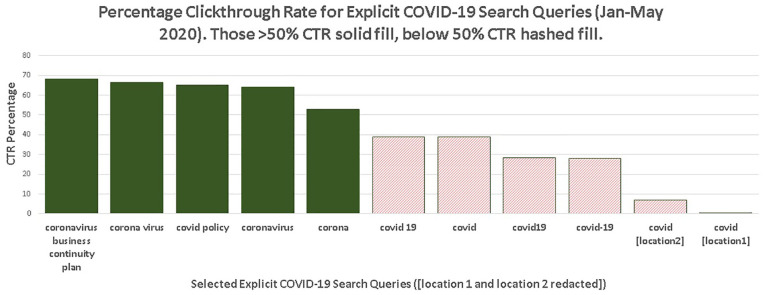
Click through rate (CTR) for explicit COVID-19 search queries (January to May 2020).

The CTR for explicit COVID-19 search queries is summarised in Figure 10 with some information redacted so the organisation cannot be identified. The organisation created numerous COVID-19 communication pages on their Intranet similar to how other organisations have behaved in a crisis [27]. However, none were promoted as the ‘key ones’ and the search engine often returned older results ranked higher than the latest information.

The search queries in Figure 10 (solid fill) have comparable CTRs to Google [51]. However, it is also clear from Figure 10 that certain COVID-19 explicit queries (hashed fill) have extremely low CTRs. For example, for the redacted search query ‘covid [location 1]’, the search query was made 129 times, but only elicited a single click. It is probable that information needs were not met.

While the organisation published content on their Intranet regarding the pandemic, search logs were not monitored by the organisation (not unusual in the enterprise [32]). This resulted in its inability to ‘know’ that uncertainty was not being made clearer in many situations. The organisation did not follow surveillance techniques assessing the impact of communications through search log analysis enabling it to make interventions [47].

Explicit COVID-19 search queries using ‘corona’ or ‘coronoavirus’ or ‘corona virus’ have higher CTRs than those with ‘covid19’, ‘covid 19’ and ‘covid’ (Figure 9). A plausible explanation is that these search queries had the same intent, but probably gave different results as synonyms were not present – the vocabulary problem [22]. The name COVID-2019 was chosen on the 11 February 2020 [11] after variations were already in use. This supports the assertion that use of synonyms may improve results and CTR.

**Figure 11.**
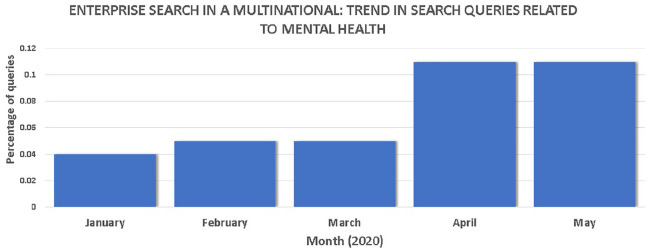
Increases in search queries over time for the ‘mental health’ topic.

In addition to explicit COVID-19 search queries, implicit search queries most probably related to COVID-19 were observed for the first time such as ‘hand washing’ and ‘travel ban’. There were also increased searches made on topics such as IT collaboration tools. Figure 11 shows a proportional increase, as a percentage of all search results made, for queries related to ‘mental health’.

This supports existing research findings [49], which reported an increase in frequency of web queries on ‘mental health’ during the lockdowns. There may be external media influence as mental health awareness week in the United Kingdom was held in late May 2020. It could also be probable that some staff may be cautious about making certain queries using their company search engine in case these can be linked back to them as individuals, which may differ to how people perceive Internet search engines, presenting an area for further research.

In summary, the enterprise search log data have yielded numerous insights for COVID-19-related search queries during a pandemic. There is a *simultaneous* narrowing of search queries by the enterprise community *in unison*, for the emerging pandemic topic. Intents have transition from single-word awareness queries to multi-word task–based queries. This emergent ordering is produced through search technology by human actors but not by human design.

**Figure 12.**
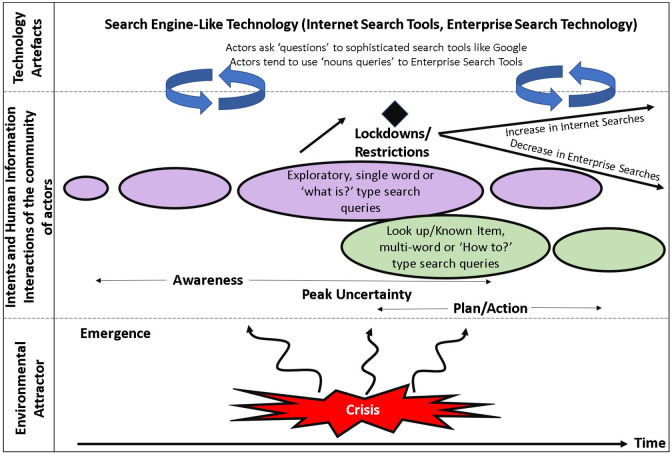
Human information interactions on a crisis topic using search engines.

Combining with the existing literature for Internet communities [52] and contrasting with traditional reductionist individual ‘user session focused’ narrowing search tactics [19–21], human information interaction regularities are observed for an entire community at the same time. This may be one of the new information interaction models supporting an ‘information crisis’ [14] and a consideration of the ecosystem, information interactions and constraints advocated by some researchers [5,6]. While the existing information science literature shows ‘narrowing’ and ‘transitioning’ of information search behaviour as primarily ‘agency’ driven, this study provides evidence for the constraining influence of ‘structure’ to also ‘narrow’ and ‘transition’ search behaviour. A new human information interaction model is proposed (Figure 12) relating to use of search technology for pandemic-related queries in an emerging crisis.

In the pre-lockdown or early part of a new crisis such as a pandemic, the community is attracted to making general awareness, exploratory, simple one word/concept exploratory search queries or broad ‘What is …’ questions from Internet searches to educate themselves. At the peak of uncertainty and work activity, a surge in information seeking is observed, with subsequent tendencies to transition to a range of lookup search task-driven intents. These spawn more specific search queries including those with more search query terms or ‘How to …’ questions, addressing preparation, impact, strategies, policies, responses and ways of working along with health concerns.

Like any case study, there may be limitations to the generalisability of findings. This study has scratched the surface of search behaviours during the COVID-19 pandemic and hopefully will act as a catalyst for more in-depth studies.

## 5. Conclusion

The move to remote working during the COVID-19 pandemic has leaned heavily on the digital infrastructure to keep people connected, informed and able to work remotely. As more staff may probably work from home more often in the future precipitated by the COVID-19 pandemic, how this may change search behaviour in the enterprise is an area for further research.

To our knowledge, this is the first published paper on the impact of a pandemic on search in an organisation. How management and staff react to such crises through the lens of the corporate search engine presents many opportunities for further research.

Thousands of search queries were made by staff in the case study organisation relating to COVID-19 to reduce uncertainty and complete work tasks. The crisis appears to act ‘like a magnet’, aligning at a general level, human information interaction within the community simultaneously in unison over an extended period. Peak ‘demand’ in terms of uncertainty and work activity seems to appear just before lockdowns, where robust remote working capability, including an effective working search engine, is probably to help support organisational resilience.

These insights may help executives, communication and health managers plan interventions. To support them, IT, knowledge and search managers can ensure synonyms are catered for, as well as supporting document-focused search queries, longer search queries and provision of appropriate content. In extreme situations such as a pandemic, it is important within enterprise search engines to return the ‘latest’ information ranked appropriately. Enabling question and answer capability may provide a better fit to certain information needs in the organisation.

Having an easy to use simple ‘Google Trends-like’ web dashboard onto the enterprise search log that captures the right things may help management and key staff in an organisation conduct trend analysis. This may allow risks to be identified quicker and, in some cases, identify risks that otherwise would not be known at all. Such as increasing volumes of staff searching on mental health topics and information needs related to the pandemic going unmet as evidenced by low CTRs.

Enterprise search logs contain the ‘digital body language’ of the community. The search logs are an under-utilised resource of intelligence that may not be exploited to the full by many organisations for monitoring business, social and health risks, and opportunities. Search logs can provide a conduit to knowledge, an epistemology for how we come to know things in our organisations. They can supplement more traditional methods and be a valuable real-time source for actionable insight in times of crisis. In extreme situations (e.g. a pandemic), companies may need to move faster, monitoring and exploiting their enterprise search transaction logs in real time as these reflect degrees of uncertainty and anxiety that may exist in the enterprise.
